# Two Unique Cases

**Published:** 1886-11

**Authors:** John Hockenhull

**Affiliations:** Cumming, Ga.


### TWO UNIQUE CASES.

BY JOHN HOCKENHULL, M. D., CUMMING, GA.

CASE I.—I was called three years ago to see Miss M—, 16 years of age, good *embonpoint*, in apparently robust health and weighing about one hundred and forty pounds. I was called to see her on account of her never having menstruated and an occlusion of the vagina having been discovered that was likely to interfere with her contemplated marriage.

The only history of symptoms that the parents could give me of her case was an uneasiness and sense of fullness in the lower part of the abdomen at such times as they thought her menstrual periods ought to have occurred. As to her emotions, passions and desire to associate with the opposite sex, her parents informed me they saw no difference between her and their other daughters. On examining the genital organs I found the external genitals normal, but within the vulva was a thick, brawny tissue that perfectly occluded the vagina. I at once passed a female catheter into the bladder and the index finger of my right hand into the rectum, and by approximating the catheter in the bladder and my finger in the rectum, I ascertained that only the walls of the bladder and the rectum were between my finger and the catheter, consequently she could not have a very deep vagina. I could feel no uterus. The young lady was engaged to marry, and I therefore determined to operate and attempt to supply what nature had failed to make. The patient was etherized, when a cut in the median line of the vulva some inch and a half until I had cut through the dense fibrous tissue, after which I broke up the cellular tissue for about an inch with my finger. I introduced into the wound a glass tube, which kept it fully distended until it healed, which it did in some three weeks. The young lady married the object of her choice. They have been living together over two years, and a few weeks ago I inquired of the father in regard to their conjugal felicity and he informed me that he heard no complaint. She has never menstruated, and in all probability the menstrual molimen that she seemed to complain of, at what she thought regular periods, was more imaginary than real.

CASE 2.—On May the 29th of the present year Mr. C— called on me to go and see his son, about seven years of age. I found him a stout, healthy-looking boy, but his parents informed me that during the preceding day and night he had been suffering occasional tenesmus, which they thought was caused by the bowels being obstructed with rocks, the mother having extracted ten small ones from the rectum during the night. I had him placed in a prone position across his father's lap, and on introducing my finger into his rectum I found small hard stones, from the size of a pea to that of the end of the little finger, firmly impacted, forming a wall that completely obstructed and distended the rectum. I had some difficulty in dislodging the first few stones, but I soon extracted them from the rectum in such rapid succession that in a short time I had taken out one hundred and eight. He then had some natural evacuations of his bowels, the dejections being mixed with stones, and before I left three hundred and forty had been discharged. The stones were of the sizes before mentioned, of black color and a hard, flinty nature, such as are seen among sand that has been frequently subjected to the action of water. His father a few days after my visit informed

[402 stones extracted from the rectum of a boy of 7] ✓

me that the total number of stones passed were four hundred and two. He suffered no further inconvenience and is now well.

